# Health-related quality of life and associated factors in heart failure with reduced ejection fraction patients at University of Gondar Hospital, Ethiopia

**DOI:** 10.3389/fcvm.2024.1436335

**Published:** 2024-08-29

**Authors:** Daniel Belay Agonafir, Biruk Mulat Worku, Hailemaryam Alemu, Tilahun Nega Godana, Shibabaw Fentahun Bekele, Abel Andargie Berhane, Desalew Getahun Ayalew, Belete Sisay Assefa, Fikadu Alemiye Molla, Gebrehiwot Lema Legese

**Affiliations:** ^1^ Department of Internal Medicine, School of Medicine, College of Medicine and Health Sciences, Wachemo University, Hosanna, Ethiopia; ^2^ Department of Internal Medicine, School of Medicine, College of Medicine and Health Sciences, University of Gondar, Gondar, Ethiopia; ^3^ Department of Internal Medicine, School of Medicine, College of Medicine and Health Sciences, Arba Minch University, Arba Minch, Ethiopia

**Keywords:** quality of life, heart failure, reduced ejection fraction, associated factors, Ethiopia

## Abstract

**Introduction:**

Living with heart failure poses challenges due to its poor prognosis and impact on quality of life, making it crucial to assess how it affects patients for better patient-centered management. This study aimed to assess quality of life and associated factors in heart failure with reduced ejection fraction patients at University of Gondar Comprehensive Specialized Hospital in Ethiopia, 2023.

**Methods:**

An “institution-based” cross-sectional study was conducted at the University of Gondar Comprehensive Specialised Hospital. The data were collected using an interviewer-administered questionnaire. Health-related quality of life was measured using the Minnesota Living with Heart Failure Questionnaire. Sociodemographic, behavioral, clinical, biochemical, and echocardiographic characteristics were included in the questionnaire. The collected data were entered into EpiData version 4.6 and exported into SPSS version 25 for analysis. Multiple linear regression analysis (*p* < 0.05) was used to measure the degree of association between quality of life and independent variables.

**Results:**

A total of 240 patients with heart failure and reduced ejection fraction participated in the study. The health-related quality of life scores for the physical, emotional, and total were 17.60 ± 10.33, 10.58 ± 6.33 and 46.12 ± 26.06, respectively. Health-related quality of life was significantly associated with age, marital status, occupation, income, heart failure duration, recent hospitalization, New York Heart Association functional class, heart failure etiology, atrial fibrillation comorbidity, systolic blood pressure, heart rate, heart failure medications, severe left ventricular systolic dysfunction, and severe or moderate pulmonary hypertension.

**Conclusion:**

This study found that patients with heart failure and reduced ejection fraction had poor health-related quality of life, influenced by identified factors. These findings aid professionals in assessing and identifying interventions that improve these patients’ quality of life.

## Introduction

Heart failure (HF) is a global public health problem with an estimated 64.3 million people suffering worldwide (1), and approximately 50% of cases are Heart Failure with reduced Ejection Fraction (HFrEF) (2). It remains a significant public health issue in many countries in Sub-Saharan Africa and is one of the top five causes of mortality in Ethiopia (3).

Health-related quality of life (HRQoL) is an assessment of how an individual's well-being may be affected over time by a disease, disability, or disorder (4, 5). It is important to evaluate the impact of a disease and the effect of medical intervention as perceived by the patient through the effects on the domains of life: physical, social, psychological and environmental (6).

Health-related quality of life in patients with HF is an important patient-reported health status measure that evaluates the impact of HF and its medical interventions on various domains of life. It is an outcome measurement tool that has recently received attention for assessing the impact of HFrEF and its treatment and is one of the primary goals of HFrEF treatment guidelines (7, 8). Despite current studies indicating HF patients’ preferences for improved HRQoL over survival, the importance of QoL is underscored in the management of patients with heart failure (9, 10).

Living with HF is challenging due to its poor prognosis and increased socioeconomic burden resulting from higher healthcare costs, debilitating symptoms such as dyspnea, edema, fatigue, and sleep disturbance, and frequent hospitalizations, all of which significantly impact HRQoL. Various studies have shown that QoL is generally poor in patients with HF and particularly in HFrEF patients (11–15). Despite limited and conflicting data comparing HRQoL among HF patients across the left ventricle ejection fraction (LVEF) spectrum, most evidence supports that HFrEF patients have a significantly affected QoL compared to other subgroups of heart failure patients (14, 16).

Poor HRQoL correlates with increased hospitalization and mortality rates, as well as higher costs imposed on health systems, families, and patients (17–19).

Different studies have identified determinant factors of HRQoL among patients with HF, most of which differ across the LVEF spectra. Being a younger woman, having low income, longer disease duration and higher NYHA functional class are associated with poor HRQoL in HF patients in general (11, 20, 21). Being an older woman, higher NYHA functional class, recent hospitalizations, having lower LVEF, lower systolic blood pressure and comorbidities are associated with poor HRQoL in HFrEF in particular (13, 14, 16, 22).

Measuring HRQoL will help monitor treatment guidelines and improve patient HRQOL. Analysis of HRQoL can identify groups with poor HRQoL and its determinant factors, guiding interventions to improve their situation and prevent more serious consequences, allocate limited resources based on unmet needs, guide strategic plans, and monitor interventions (7, 8).

Although the impact of HF on QoL and its determinants differs across the LVEF spectrum, as evidenced by available studies, there is a scarcity of studies on subgroups of HF populations. Therefore, this study aimed to assess HRQoL and associated factors among adult patients with HFrEF.

## Methods and materials

### Study design and settings

The study was a cross-sectional “institutional-based” study conducted from August to November 2023 at the University of Gondar Comprehensive Specialized Hospital (UoGCSH) in North West Ethiopia. It is one of the largest hospitals in the country, providing health services to around 13 million inhabitants in its catchment area. The hospital has various follow-up clinics for major chronic illnesses, including the Cardiac clinic. Approximately 3,000 cardiac patients receive follow-up care at this clinic.

### Population

All adult patients with HFrEF who had chronic follow-up at UoGCSH were source population. The study population consisted of all adult patients with HFrEF who visited the chronic follow-up clinic at UoGCSH during the study period.

### Inclusion and exclusion criteria

Adult patients (aged 18 years or older) with HFrEF who had been followed up for at least three months at the cardiac clinic at UoGCSH were included in the study.

Patients with cognitive impairment, such as dementia or psychosis (who were not likely to recall their conditions well enough), concomitant acute illnesses, such as Acute HF exacerbation, severe chest infections, or other infections that could acutely influence their QoL, and coexisting diagnosis of other chronic illnesses (end-stage renal disease, decompensated chronic liver disease, chronic obstructive pulmonary disease, cerebrovascular accident, and diabetes mellitus) were excluded from the study.

### Sample size and sampling procedure

The sample size was calculated based on the mean estimate from previous research conducted in Brazil (22) using the mean estimation formula. The final sample size was determined to be 253 after adding a 10% non-response rate.

A consecutive sampling technique was employed, in which every participant who met the inclusion criteria was selected until the desired sample size was reached.

### Data collection tools and procedures

The data was collected using structured questionnaires. The questionnaire included sociodemographic, behavioral, clinical, biochemical, and echocardiographic parameters, as well as the Minnesota Living with Heart Failure Questionnaire (MLHFQ) tool.

The MLHFQ is one of the most widely used disease specific HRQoL questionnaires for patients with HF and has been validated in many countries worldwide (23–25). It provides scores for two dimensions, physical and emotional, as well as a total score. HF-specific health status assessments are preferred because they are more sensitive to changes in disease status and more responsive to HF therapy than generic health status measures (26–28).

The English version of the MLHFQ was translated into Amharic, a local language, and then back into English by another person to ensure consistency. Data related to clinical, biochemical, and echocardiographic parameters were obtained by reviewing patients’ medical charts. Concerning the biochemical parameters, specifically Hemoglobin, Serum creatinine, Glomerular filtration rate, and Serum sodium, the average of the laboratory results obtained during the three months preceding the patient's visit is utilized. Laboratories were done via Yumizen H550 automated hematology analyzer.and AU480 chemistry analyzer. Sociodemographic, behavioral variables, and all 21 MLHFQ variables were obtained by trained interviewers who conducted face-to-face interviews. These interviewers were two nurses with experience working in a cardiac clinic service, and a general practitioner was recruited as a supervisor.

To ensure data quality, all data collectors and supervisors were trained in data collection procedures, the objective and relevance of the study, and the confidentiality of information. The Amharic version of the MLHFQ was used to ensure that study participants could easily understand it. Pretest data were collected from 15 study participants at the UoGCSH follow-up clinic to check reliability, wording, and identify any language barriers and contextual variations. During data collection, accuracy and completeness were checked daily by the supervisor and the principal investigator.

### Study variables

#### Dependent variable

Health Related Quality of Life (Physical, Emotional and Total HRQoL).

#### Independent variables

Socio-demographic: age, sex, residency, marital status, educational level, occupation and income.

Behavioral: Current smoking, alcohol and salt intake.

Clinical: Systolic blood pressure (SBP), Heart rate (HR), Duration of HF, etiology of HF (IHD, DCMP, CRVHD, DVHD, others), NYHA functional class (I—IV), Recent hospitalization in the past 6 months, Co-morbidities (atrial fibrillation, hypertension, anemia, obesity, dyslipidemia, others), HF related medications (Diuretics, B-blockers, ACEI/ARBs, Spironolactone, Digoxin, Antiplatelets, Anticoagulants, Statin, others).

Biochemical: Hemoglobin, Serum creatinine, Glomerular filtration rate, Serum sodium (mean of results obtained within the past 3 months).

Echocardiographic: Etiology of HF, LVEF, and severity of pulmonary hypertension on baseline echocardiography (echo) performed at the time of HFrEF diagnosis.

### Operational definitions

#### Heart failure with reduced ejection fraction patients

Heart failure patients who have echocardiographic measurement of left ventricular ejection fraction ≤40% labelled as HFrEF patients.

#### Health-related quality of life

Health-Related Quality of Life serves as an outcome variable assessed through the MLHFQ instrument. This tool comprises 21 items specifically designed to evaluate HRQoL in relation to a particular disease, prompting participants to reflect on the impact of their condition over the past month. Each item is rated on a six-point Likert scale, ranging from 0 (indicating no effect) to 5 (indicating a significant effect). The individual responses are aggregated to form a composite score that reflects the overall HRQoL of the participants. Scores can vary from 0 to 105, with higher scores indicating a lower quality of life. The instrument yields a total score along with scores across two dimensions: physical (covering questions 2–7, 12, and 13, with a range of 0–40) and emotional (covering questions 17–21, with a range of 0–25). The remaining eight questions (1, 8, 9, 10, 11, 14, 15, and 16) contribute solely to the calculation of the total composite score. Heart failure patients are categorized based on their total HRQoL scores, with those scoring below 24 classified as having “Good” HRQoL, scores between 24 and 45 as “Moderate,” and scores exceeding 45 as “Poor” HRQoL (29).

#### New York Heart Association Functional Class

Used to classify the severity of heart failure as.

Class I: no limitation during ordinary activity, Class II: slight limitation during ordinary activity, Class III: marked limitation of normal activities without symptoms at rest, Class IV: unable to undertake physical activity without symptoms and symptoms may be present at rest (30).

### Data processing and analysis

The completeness of the collected data was checked. Next, codes were assigned to each question and entered into EpiData version 4.6. The data was then exported to statistical software SPSS version 25 for further analysis. A reliability test (Cronbach's alpha) was conducted to assess the reliability of the items in the MLHFQ tool. The Cronbach's alpha was 0.99 for total HRQoL, 0.98 for the physical domain and also 0.98 for the emotional domain. Descriptive analyses such as frequencies, means and medians were conducted for the outcome and independent variables. In cases were an item was missing, the mean of the other items in the domain was used as a substitute.

The health-related quality of life, as measured by the MLHFQ tool, was outcome variable, consisting of three domains (physical, emotional and total HRQoL). Socio-demographic, behavioral, clinical, biochemical and echocardiographic variables were utilized as independent variables.

Bivariate analysis for categorical variables was performed using independent-samples *t*-test or one-way ANOVA to examine differences in mean MLHFQ score among the categories. For continuous variables, bivariate analysis using Pearson's correlation was conducted to assess their correlation with HRQoL.

Variables that showed an association with HRQoL at a significance level of *p* < 0.25 during bivariate analysis were included in the multivariate analysis.

Multiple linear regression analysis using a stepwise regression model was carried out for the multivariate analysis of the association between the HRQoL domains and demographic, behavioral, clinical, biochemical and echocardiographic variables.

Model assumptions (linearity, multicollinearity, independence, equal variance, normality and influential case biasing) were verified.

Variables with a *p*-value < 0.05 after multiple linear regression were deemed statistically significant.

## Results

### Sociodemographic characteristics of study participants

A total of 240 patients with heart failure with reduced ejection fraction, who were receiving follow-up at a cardiac clinic, participated in the study with a response rate of 94.9%. The mean age of the study participants was 51.04 ± 13.2 years. Among the participants, 52.1% were female, 50.8% lived in urban areas, 61.3% were married, 34.2% were housewives, and 42.9% had no formal education. The mean monthly income was 3,473.3 ± 1,605.4 Ethiopian Birr (ETB).

In this study, 9.2% of participants were current smokers, 33.3% reported salt intake and 19.2% were currently drinking alcohol (Table 1).

**Table 1:** Socio-Demographic and behavioral characteristics of patients with heart failure with reduced ejection fraction attending cardiac clinic at UoGCSH North West Ethiopia, 2023 (*n* = 240).

Variables	Category	Frequency	Percent
Age	Mean ± SD 51.04 ± 13.22		
Sex	Male	115	47.9
	Female	125	52.1
Residence	Rural	118	49.2
	Urban	122	50.8
Marital status	Married	147	61.3
	Single	29	12.1
	Divorced	29	12.1
	Widowed	35	14.6
Occupation	Farmer	61	25.4
	Merchant	52	21.7
	Housewife	82	34.2
	Government & nongovernment employee	45	18.8
Level of education	No formal education	103	42.9
	Primary school	53	22.1
	Secondary school	39	16.3
	College and above	45	18.8
Monthly income (ETB)	Mean ± SD 3,473.3 ± 1,605.4		
Smoking	No	218	90.8
	Yes	22	9.2
Alcohol	No	194	80.8
	Yes	46	19.2
Salt intake	Yes	80	33.3
	No	160	66.7

### Clinical characteristics of study participants

The median (IQR) SBP of study participants was 105(20) mmHg ranging from 75 to 150 mmHg while the median (IQR) HR was 89(23) bpm ranging from 40 to 112 bpm. The median (IQR) duration of illness was 1.5 (1.0) year, ranging from 6 months to 6 years. The majority (61.7%) of the participants were in NYHA class III and IV, and 35.8% had a history of recent admission within the past 6 months. Etiologies of HFrEF identified among respondents were IHD (39.2%), CRVHD (25.8%), DCMP (23.8%) and DVHD (11.3%). In this study 61.7% of participants had at least one of the comorbidities. AF (25.4%), HTN (23.3%), Anemia (21.7%), Dyslipidemia (18.3%), Thyrotoxicosis (10.4%) and Obesity (8.8%) were among the identified comorbidities. Participants were taking medications including ACEI/ARB (91.3%), Beta blockers (77.1%), Diuretics: furosemide (52.5%), Statin (51.2%), Antiplatelets (40.4%), Spironolactone (32.9%), Anticoagulants (25.4%), Anti thyroid (10%), Digoxin (5%) and Antibiotics (5%) (Table 2).

**Table 2:** Clinical characteristics of patients with heart failure with reduced ejection fraction attending cardiac clinic at UoGCSH North West Ethiopia, 2023 (*n* = 240).

Variables	Category	Frequency	Percent
Systolic BP (mmHg)	Median (IQR) 105 (20)		
Heart rate (bpm)	Median (IQR) 89 (23)		
Duration of HFrEF (years)	Median (IQR) 1.5 (1)		
NYHA class	I	32	13.3
	II	60	25.0
	III	107	44.6
	IV	41	17.1
Hospitalization within 6 months	No	154	64.2
	Yes	86	35.8
Etiology of HFrEF	IHD	94	39.2
	DCMP	57	23.8
	CRVHD	62	25.8
	DVHD	27	11.3
Comorbidities	No	79	32.9
	Yes	161	67.1
Anemia	No	188	78.3
	Yes	52	21.7
Hypertension	No	184	76.7
	Yes	56	23.3
Atrial fibrillation	No	179	74.6
	Yes	61	25.4
Thyrotoxicosis	No	215	89.6
	Yes	25	10.4
Obesity	No	219	91.2
	Yes	21	8.8
Dyslipidaemia	No	196	81.7
	Yes	44	18.3
Other comorbidities	No	227	94.6
	Yes	13	5.4
Medication			
ACEIs/ARBs	No	21	8.7
	Yes	219	91.3
b-Blockers	No	55	22.9
	Yes	185	77.1
Spironolactone	No	161	67.1
	Yes	79	32.9
Diuretics (furosemide)	No	114	47.5
	Yes	126	52.5
Digoxin	No	228	95.0
	Yes	12	5.0
Antiplatelets	No	143	59.6
	Yes	97	40.4
Anticoagulants	No	179	74.6
	Yes	61	25.4
Statins	No	117	48.8
	Yes	123	51.2
Antibiotics	No	228	95.0
	Yes	12	5.0
AntiThyroids	No	216	90.0
	Yes	24	10.0
Others	No	235	97.9
	Yes	5	2.1

### Biochemical and echocardiographic characteristics of study participants

In this study, the mean values of laboratory results conducted within the past 3 months were included. The median (IQR) value of Hg was 14.1(2) g/dl ranging from 9.5 to 18.3 g/dl and Cr was 0.89(0.23) mg/dl ranging from 0.45 to 1.80 mg/dl. The mean value of eGFR was 92.48 ± 20.52 ml/min/1.73 m^2^ and Na was 136.22 ± 4.70 mEq/L.

The median (IQR) of baseline LVEF was 30(7.75)%, ranging from 10% to 40%, 36.3% had severe systolic dysfunction, and 63.7% had moderate systolic dysfunction. 62.5% had mild or no PH, while 37.5% had severe or moderate pulmonary hypertension (PH) on baseline echocardiography (Table 3).

**Table 3:** Biochemical and echocardiographic characteristics of patients with heart failure with reduced ejection fraction attending cardiac clinic at UoGCSH North West Ethiopia, 2023 (*n* = 240).

Variables	Category	Frequency	Percentage
Hemoglobin, g/dl	Median (IQR) 14.1 (2)		
Creatinine, mg/dl	Median (IQR) 0.89 (0.23)		
Glomerular filtration rate, ml/min/1.73 m^2^	Mean ± SD = 92.48 ± 20.52		
Serum sodium, mEq/L	Mean ± SD = 136.22 ± 4.70		
Baseline echocardiography LVEF, %	Median (IQR) 30 (7.75)		
Baseline echocardiography LVEF class	From 30% to 40% (Moderate systolic dysfunction)	153	63.7
	Less than 30% (sever systolic dysfunction)	87	36.3
Baseline echocardiography pulmonary hypertension severity	Mild or none	150	62.5
	Moderate or sever	90	37.5

### Health-related quality of life of heart failure with reduced ejection fraction patients

In this study the MLHFQ assessed HRQoL and showed mean scores of 17.60 ± 10.33, 10.58 ± 6.33 and 46.12 ± 26.06 for physical, emotional and total QoL respectively. Approximately 43.3% of the participants were assessed as having a poor quality of life related to heart failure, while the remaining 29.2% and 27.5% were assessed as having moderate and good HRQoL, respectively (Table 4).

**Table 4:** Health-Related quality of life of patients with heart failure with reduced ejection fraction attending cardiac clinic at UoGCSH north west Ethiopia, 2023 (*n* = 240).

HRQoL domains	Mean ± SD	95% confidence interval	Cronbach's Alpha
Physical (range 0–40)	17.60 ± 10.33	16.28–18.90	0.98
Emotional (range 0–25)	10.58 ± 6.33	9.77–11.38	0.98
Total (range 0–105)	46.12 ± 26.06	42.80–49.43	0.99
Level of total HRQoL	Frequency	Percent	
Good (score 0–23)	66	27.5	
Moderate(score 24–45)	70	29.2	
Poor (score 46–105)	104	43.3	

### Factors associated with health-related quality of life

Multivariate analysis was conducted using a multiple linear regression model, with categorical variable (after conversion to dummy variables) and continuous variables as independent variables, and HRQoL domains (physical, emotional, total QoL) as outcome variables.

The analysis revealed that the total HRQoL domain was significantly associated with Age, Marital status, Occupation, Monthly income, SBP, HR, Duration of HF, NYHA class, Recent Hospitalization, Etiology of HF, AF comorbidity, Anticoagulant and furosemide use as HF medications, Baseline echo systolic dysfunction and PH severity. The final R-square value was 0.879, indicating that 87.9% of the variation in total HRQoL was explained by the identified associated factors.

As age increased, the scores of physical and total HRQoL domains also increased. Specifically, for every one-year increase in age, the total HRQoL score increased by 0.37 (95% CI = 0.24, 0.50), and the physical domain HRQoL score increased by 0.15 (95% CI = 0.10, 0.20).

Compared to married individuals, single and widowed participants had higher HRQoL scores in all domains. Employed respondents (both governmental and non-governmental) had higher physical and total HRQoL scores compared to housewives, while farmers had only a higher physical HRQoL score.

As income increased, the score of all HRQoL domains decreased. For every 1000ETB increase in monthly income, the total HRQoL score decreased by 2.00 (95% CI = −2.20, −1.20), the physical HRQoL score decreased by 2.00 (95% CI = −3.20, −1.20), and the emotional HRQoL score decreased by 3.00 (95% CI = −5.10, −1.10).

Similarly as SBP increased, the score of all HRQoL domains decreased. For every one mmHg increase in SBP, the total HRQoL score decreased by 0.15 (95% CI = −0.27,−0.04), the physical domain HRQoL score decreased by 0.06 (95% CI = −0.11, −0.01), and the emotional domain HRQoL score decreased by 0.08 (95% CI = −0.12, −0.05).

As HR increased, the score of physical and total HRQoL domains increased. For every one bpm increase in HR, the total HRQoL score increased by 0.19 (95% CI = 0.07, 0.31), and the physical domain HRQoL score increased by 0.11 (95% CI = 0.06, 0.15).

An increase in the duration of HFrEF was associated with a decrease in total HRQoL domain score. Specifically, for every one-year increase in duration, the total HRQoL score decreased by 1.73 (95% CI = −3.05, −0.40).

Participants in NYHA class-III and class-IV had higher HRQoL scores in all domains compared to those in NYHA class I and II. Participants in NYHA class-III had an increased total HRQoL score by 6.39 (95% CI = 3.04, 9.74), an increased physical HRQoL score by 3.02 (95% CI = 1.65, 4.40) and increased emotional HRQoL score by 1.36 (95% CI = 0.36, 2.37). Participants in NYHA class-IV had an increased total HRQoL score by 17.89 (95% CI = 12.57, 23.20), an increased physical HRQoL score by 6.86 (95% CI = 4.69, 9.03) and an increased emotional HRQoL score by 4.55 (95% CI = 3.02, 6.09).

Respondents who were recently hospitalized had an increased total HRQoL score by 8.56 (95% CI = 4.38, 12.74), an increased physical domain HRQoL score by 4.21 (95% CI = 2.49, 5.93), and an increased emotional HRQoL score by 2.29 (95% CI = 0.99, 3.58) compared to those with no history of admission.

Participants with DCMP as the etiology of HFrEF had an increased total HRQoL score compared to those with CRVHD, while those with DVHD had a higher emotional HRQoL score.

Participants with AF as a comorbidity had an increased total HRQoL score by 8.11 (95% CI = 4.26, 11.97), an increased physical domain HRQoL score by 2.07 (95% CI = 0.50, 3.64) and an increased emotional HRQoL domain by 2.08 (95% CI = 1.03, 3.13) compared to those without AF.

Participants using diuretics (furosemide) and anticoagulant medication had increased HRQoL score in all domains compared to those not using them.

Respondents with LV severe systolic dysfunction at baseline echocardiography had an increased total HRQoL score by 3.41(95% CI = 0.38, 6.43) compared to those with moderate LV systolic dysfunction.

Respondents with moderate or severe pulmonary hypertension had an increased total HRQoL score by 7.76 (95% CI = 3.47, 12.04), an increased physical HRQoL score by 2.84 (95% CI = 1.10, 4.59) and an increased emotional HRQoL score by 2.47 (95% CI = 1.19, 3.75).

**Figure 1:**
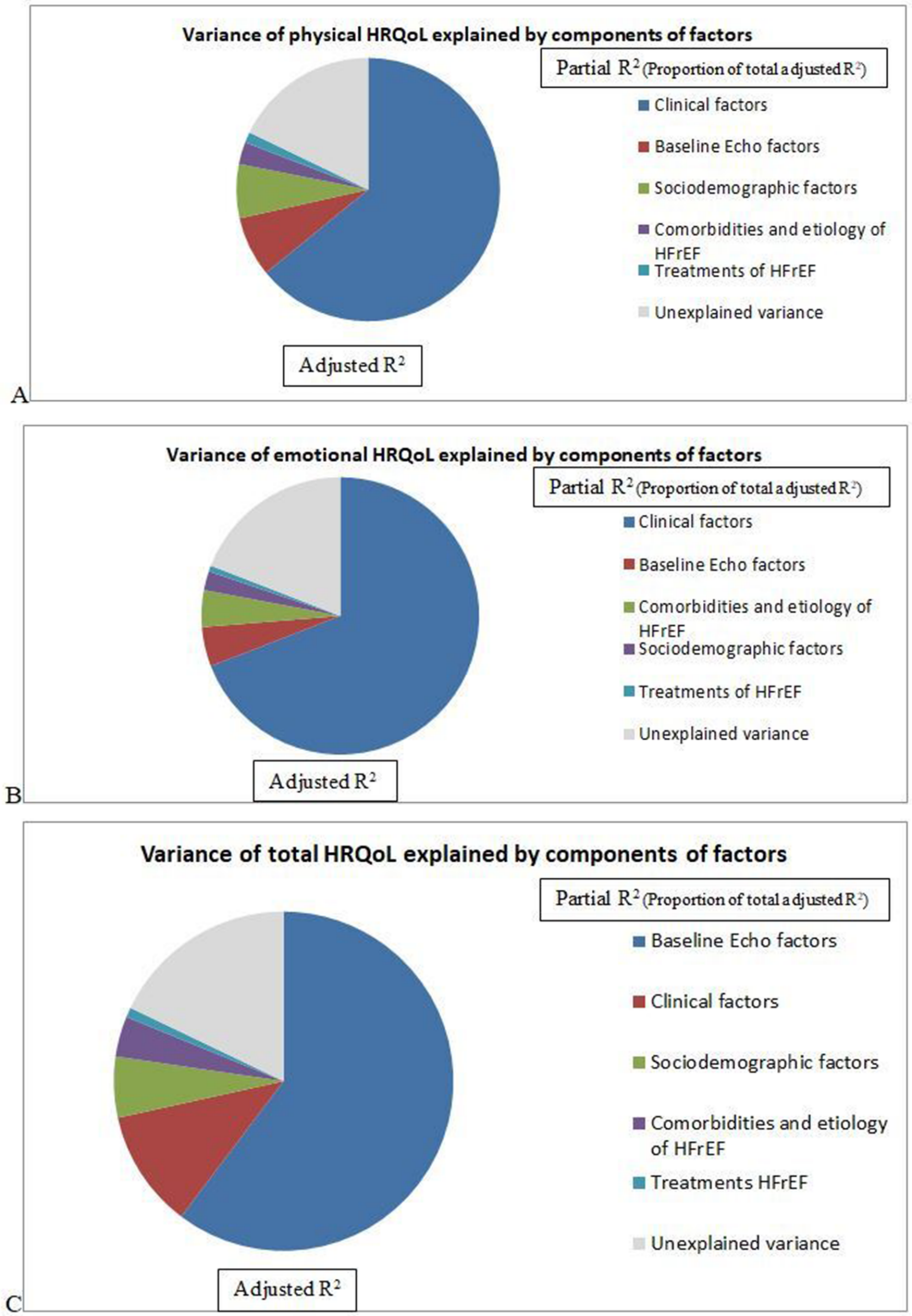
Variance of HRQoL **(A)** physical, **(B)** emotional, **(C)** total HRQoL) explained by groups of factors among patients with heart failure with reduced ejection attending cardiac clinic at UoGCSH North West Ethiopia, 2023 (*n* = 240).

The total adjusted R^2^ in this model was 0.879 for total HRQoL. Most of the significant correlates of total HRQoL were related to echocardiographic factors (partial R^2^ = 0.644) followed by Clinical factors (partial R^2^ = 0.120), Sociodemographic factors (partial R^2^ = 0.062), Comorbidity and etiology related factors (partial R^2^ = 0.041), and Treatment related factors (partial R^2^ = 0.010) (Table 5 and Figure 1).

**Table 5:** Factors associated with health-related quality of life among patients with heart failure with reduced ejection fraction attending cardiac clinic at UoGCSH, northwest Ethiopia, 2023 (*n* = 240).

Variable	Physical-HRQoL			Emotional-HRQoL			Total-HRQoL		
	ß	95% CI	*P*-value	ß	95% CI	*P*-value	ß	95% CI	*P*-value
Age	0.15	(0.10, 0.20)***	0.000	0.55		0.174	0.37	(0.24, 0.50)***	0.000
Marital status									
Married (ref)	–	–	–	–		–	–	–	–
Single	3.45	(1.84, 5.06)***	0.000	0.032		0.297	8.67	(4.66, 12.68)***	0.000
Divorced	0.027		0.264	0.014		0.637	0.045		0.062
Widowed	2.45	(1.00, 3.90)**	0.001	1.36	(0.33, 2.40)**	0.010	5.46	(1.94, 8.98)**	0.003
Occupation									
Housewife (ref)	–	–	–	–		–	–	–	–
Farmer	1.83	(0.49, 3.17)**	0.008	−0.034		0.320	−0.008		0.752
Merchant	0.025		0.384	0.017		0.657	0.042		0.130
Employee	1.83	(0.81, 1.24)**	0.008	−0.010		0.848	5.79	(2.53, 9.05)**	0.001
Education level									
Noformal edu (ref)	–		–	–	–	–	–		
Primary edu	−0.004		0.877	−0.033		0.312	−0.016		0.498
Secondary edu	0.014		0.563	1.27	(0.22, 2.32)*	0.018	0.035		0.146
College & above	0.016		0.717	2.21	(1.08, 3.33)***	0.000	0.031		0.478
Monthly income	−0.002	(−0.0003, −0.001)*	0.032	−0.003	(−0.005, −0.001)***	0.000	−0.002	(−0.002, −0.001)**	0.001
Smokers	0.022		0.361	1.64	(0.34, 2.93)*	0.013	0.038		0.109
Systolic BP	−0.06	(−0.11, −0.01)*	0.020	−0.08	(−0.12, −0.05)***	0.000	−0.15	(−0.27, −0.04)*	0.011
Heart rate	0.11	(0.06, 0.15)***	0.000	0.044	0.069	0.083	0.19	(0.07, 0.31)**	0.002
Duration of HF	−0.051		0.061	−0.021		0.503	−1.73	(−3.05, −0.40)*	0.011
NYHA class									
I & II (ref)	–	–	–	–	–	–	–	–	–
III	3.02	(1.65, 4.40)***	0.000	1.36	(0.36, 2.37)***	0.008	6.39	(3.04, 9.74)***	0.000
IV	6.86	(4.69, 9.03)***	0.000	4.55	(3.02, 6.09)***	0.000	17.89	(12.57, 23.20)***	0.000
Recent hospitaliz									
No (ref)	–	–	–	–	–	–	–	–	–
Yes	4.21	(2.49, 5.93)***	0.000	2.29	(0.99, 3.58)**	0.006	8.56	(4.38, 12.74)***	0.000
Etiology									
CRVHD (ref)	–		–	–	–	–	–	–	–
IHD	−0.027		0.363	0.025		0.537	0.019		0.621
DCMP	0.042		0.087	1.11	(0.25, 1.98)*	0.012	3.20	(0.34, 6.07)*	0.029
DVHD	0.023		0.369	1.74	(0.54, 2.94)**	0.005	0.024		0.392
Atrial fibrillation									
No (ref)	–	–	–	–	–	–	–	–	–
Yes	2.07	(0.50, 3.64)*	0.010	2.08	(1.03, 3.13)***	0.000	8.11	(4.26, 11.97)***	0.000
Diuretics(furosemide)									
No (ref)	–	–	–	–	–	–	–	–	–
Yes	2.98	(1.82, 4.15)***	0.000	1.44	(0.57, 2.32)**	0.001	6.70	(3.88, 9.52)***	0.000
Anti-coagulants									
No (ref)	–	–	–	–		–	–	–	–
Yes	1.4	(0.15, 2.65)*	0.028	0.029		0.379	3.04	(0.01, 6.07)*	0.049
Baseline echo EF									
Moderate (ref)	–		–	–		–	–	–	–
Severe	0.051		0.083	0.011		0.761	3.41	(0.38, 6.43)*	0.027
Baseline echo PH									
Mild/none (ref)	–	–	–	–	–	–	–	–	–
Moderate/Sever	2.84	(1.10, 4.59)**	0.002	2.47	(1.19, 3.75)***	0.000	7.76	(3.47, 12.04)***	0.000
Adjusted-R^2^	0.872			0.809			0.879		

ß, unstandardized regression coefficient; CI, confidence interval; ref, reference group.

* Variables significant with *p* value <0.05.

** Variables significant with <0.01.

*** Variables significant with *p* value <0.001.

## Discussion

This study aimed to assess the HRQoL and associated factors among HFrEF patients. The findings from this study show that HFrEF has a significant impact on the HRQoL of patients. Socio-demographic factors such as age, marital status, occupation, education level and income; clinical factors such as SBP, HR, duration of HF, NYHA functional class, recent admission; etiology and comorbidity of HF such as AF and DCMP; HF medications including diuretics (furosemide) and anticoagulants; and echocardiographic factors such as baseline LVEF and PH severity had a significant association with total HRQoL.

The mean total HRQoL score was 46.12, indicating that patients with HFrEF have poor HRQoL. This finding is comparable to previous studies conducted in low-middle income countries like Tunisia and Albania, which reported scores of 41.5 and 50 respectively (31, 32).

The mean total HRQoL score of the current study was higher than similar studies conducted in Brazil (34.9) (22) and Thailand (32.4) (33) using the same measurement tool (MLHFQ), indicating better HRQoL. This could be attributed to differences in the study population and the quality of health care services in these countries.

The mean total HRQoL score in the current study was lower than those reported in studies conducted in Vietnam and China, which had scores of 67.19 and 74.16 respectively, indicating a poorer HRQoL (34, 35). This difference could be attributed to variations in the study population and sample size, which were smaller in both of those studies.

The current study identified various socio-demographic factors influencing the HRQoL of patients with HFrEF.

Increasing age was significantly associated with an increased HRQoL score (poor HRQoL), supported by similar studies showing a decline in HRQoL as people age (13, 36, 37). This finding can be attributed to the fact that older individuals experience more physical limitations and psychological problems.

Compared to married individuals, single and windowed individuals had poorer HRQoL, possibly due to a lack of support from their partners (38).

Employed participants and those with a secondary education level or higher also has poorer HRQoL, consistent with previous study by Audi G et al. (20). This finding could be due to emotional instability and higher expectations in their life among educated though being employed.

Participants with lower monthly incomes had poorer HRQoL supported by previous studies (37, 39, 40), likely due to financial hardships related to medication and healthcare costs impacting HF management and HRQoL.

Behavioral and lifestyle characteristics of the study participants did not show a significant association with HRQoL like that of other studies participants (14, 31), except for smoking, which was associated with poor emotional HRQoL, likely due to increased anxiety and tension among smokers. Studies showed a negative effect of salt enriched diet and a positive effect of exercise on quality of life in heart failure patients (11, 20). This may be salt/sodium results in fluid retention which will lead to acute exacerbation of heart failure, and regular exercise leads to muscle strengthening and improvement of mental and physical disease.

Several clinical factors influencing the HRQoL of people with HFrEF were identified in this study.

An increase in HR and a decrease in SBP were significantly associated with increased HRQoL score (poor HRQoL) supported by previous study (41), possibly indicating an advanced HF state.

The duration of HF had a negative relationship with HRQoL, supported by study by Paz LFA et al. (2019) that showed patients with less than a year in the service had worse HRQoL (42). This can be due to patient's get less time to adapt to the consequences of this chronic illness.

NYHA functional classes were significantly associated with all domains of HRQoL, with patients in classes III and IV having poorer HRQoL, consistent with previous studies (13, 14, 31, 32, 40, 43). This is because symptom distress affects patients’ everyday lives with heart failure and lead to a diminished HRQoL (44).

Recent admissions were associated with reduced physical, emotional and total HRQoL, similar to the finding from Spain and Brazil (13, 43), likely reflecting disease burden, disease progression, and poor HRQoL.

DCMP was associated with poorer HRQoL, also shown in other study (45), likely due to severely depressed systolic function. Patients with DVHD had lower emotional HRQoL, possibly due to the older age of these patients.

Atrial fibrillation as a comorbidity was associated with poor HRQoL, likely due to symptom distress (46, 47).

The use of diuretics (furosemide) and anticoagulant was associated with poor HRQoL, possibly due to disease progression and the need for frequent monitoring (40, 48, 49).

Severe left ventricular systolic dysfunction and moderate/severe pulmonary hypertension were associated with poor HRQoL, likely indicating disease advancement. These findings are consistent with studies in Poland (14), Saudi Arabia (50) and Brazil (43).

## Strength and limitations of the study

The findings provide valuable insights for clinical practice, aiding in the development of targeted interventions to improve HRQoL in HFrEF patients.

However, this study has limitations that should be considered when interpreting its results.

The cross-sectional design limits causal inferences, highlighting the need for longitudinal studies. Additionally, the reliance on self-reported data may introduce recall and social desirability biases.

## Conclusion

Heart failure with reduced ejection fraction has a significant impact on patients’ HRQoL. In this study, the majority of HFrEF patients were found to have lower HRQoL. Factors such as older age, being single or widowed, employment status, lower income, lower SBP, higher HR, shorter duration of HF, higher NYHA classes, recent hospitalization, DCMP as the etiology of HF, AF as a comorbidity of HF, medication use (furosemide & anticoagulants), severe LV systolic dysfunction, and severe/moderate PH were all associated with poor HRQoL.

This finding underscores the importance of including HRQoL as an additional measure when evaluating patients with HFrEF, as other clinical variables used to assess risk may not capture the same information as instruments that gauge the patients’ perception of health. This approach can help identify patients who require special interventions.

Providing more personalized education and adjusting medical treatments/interventions can assist patients with poor HRQoL in managing their daily lives effectively.

## Data Availability

The original contributions presented in the study are included in the article/Supplementary Material, further inquiries can be directed to the corresponding author.
